# Biased Maintenance of Attention on Sad Faces in Clinically Depressed Youth: An Eye-Tracking Study

**DOI:** 10.1007/s10578-021-01229-z

**Published:** 2021-09-02

**Authors:** Christina Buhl, Anca Sfärlea, Johanna Loechner, Kornelija Starman-Wöhrle, Elske Salemink, Gerd Schulte-Körne, Belinda Platt

**Affiliations:** 1 Department of Child and Adolescent Psychiatry, Psychosomatics and Psychotherapy, LMU University Hospital Munich, Pettenkoferstr. 8a, 80336 Munich, Germany; 2 German Youth Institute, Munich, Germany; 3 Department of Clinical Psychology, Utrecht University, Utrecht, The Netherlands

**Keywords:** Attention bias, Youth depression, Risk for depression, Eye-tracking

## Abstract

The role of negative attention biases (AB), central to cognitive models of adult depression, is yet unclear in youth depression. We investigated negative AB in depressed compared to healthy youth and tested whether AB are more pronounced in depressed than at-risk youth. Negative AB was assessed for sad and angry faces with an eye-tracking paradigm [Passive Viewing Task (PVT)] and a behavioural task [Visual Search Task (VST)], comparing three groups of 9–14-year-olds: youth with major depression (MD; n = 32), youth with depressed parents (high-risk; HR; n = 49) and youth with healthy parents (low-risk; LR; n = 42). The PVT revealed MD participants to maintain attention longer on sad faces compared to HR, but not LR participants. This AB correlated positively with depressive symptoms. The VST revealed no group differences. Our results provide preliminary evidence for a negative AB in maintenance of attention on disorder-specific emotional information in depressed compared to at-risk youth.

**Supplementary Information:**

The online version contains supplementary material available at 10.1007/s10578-021-01229-z.

## Introduction

Major Depression (MD) is among the most common mental illnesses in children and adolescents with a prevalence of 17% up to the age of 18 years [1]. It not only has as major impact on development during childhood and adolescence but it is also associated with adverse outcomes in adulthood regarding education, social life and physical [2] as well as mental [3] health. Considerable neurobiological and cognitive development as well as high neural plasticity during childhood and adolescence may explain why youth are more vulnerable to psychopathology [4, 5]. With modest effects and high recurrence rates, however, current standard treatment does not suffice [6]. In order to refine and complement existing treatment, a more detailed understanding of mechanisms underlying development and maintenance of depression in youth is needed.

### Negative Attention Biases (AB) in Depression

Cognitive models of depression consider cognitive biases a key element in development and maintenance of MD [7, 8]. Cognitive biases describe a shift in three aspects of information processing: attention to, interpretation of and memory for emotional information [9–11]. This shift promotes the filtering of incoming information according to the depressive schemas that initially activated the biases and in turn results in confirming these schemas, facilitating negative mood [8]. The shift in attention towards negative compared to positive or neutral information is referred to as negative attentional biases (AB). According to the model of vulnerability to depression by De Raedt and Koster [12], attentional processes are influenced by genetic and neuroregulatory factors [e.g. hypothalamic–pituitary–adrenal (HPA) axis functioning and impaired attentional networks] and are bi-directionally associated with emotion regulation (e.g. rumination). In this framework, negative AB would be essential in linking psychological and biological vulnerabilities for depression and its recurrence [12].

Importantly, attention is not a unitary process but comprises multiple consecutive components: orientation, maintenance and detachment of attention [13]. Studies on adult depression find reliable evidence for AB in the maintenance of attention on negative disorder-specific stimuli, while results regarding orientation of attention are less robust [11, 13]. Since major development occurs in cognitive and emotional functioning [14] and in attentional networks [15] during childhood and adolescence, results from adult studies cannot be directly transferred to youth. Cognitive patterns might not evolve into stable, trait-like “cognitive styles” until adulthood [16], therefore these might have less influence on cognitive vulnerability in youth than in adult depression. Alternately, brain maturation and hormonal changes associated with an enhanced emotional sensitivity [17] might make youth more susceptible to negative cues in ambiguous emotional information, resulting in more pronounced negative cognitive biases.

### Negative Attention Biases (AB) in Youth Depression

There is a growing body of literature on AB in depressed youth, yet findings are heterogeneous [18]: while some studies found youth with MD to show increased attention *towards* negative disorder-specific (sad) information [19, 20], another study found them to *avoid* sad stimuli [21]. An association between negative AB towards threatening information (angry faces) and depressive symptoms in unselected youth was observed by one study [22], while no evidence for AB was discovered at all in a different study [23]. These differences could be due to moderating factors like genetic differences in e.g. hypothalamic–pituitary–adrenal (HPA) axis reactivity [24], or due to the different methods employed or different aspects of attention measured (e.g. allocation, maintenance with or without distraction) in different samples. As such, the direction of AB, i.e. whether depressed individuals prefer or avoid (i.e., show AB towards or away from) negative stimuli is unclear. Results are also unequivocal regarding specificity, i.e. whether AB apply to disorder-specific stimuli (e.g. sad faces) or threatening stimuli (e.g. angry faces) or negative information in general.

### Measuring AB

To assess AB, reaction-time (RT) based behavioural measures are used regularly, with speed of response acting as indicator for AB towards or away from emotional stimuli. Investigating AB by measuring RT involves drawing indirect inferences about the focus of attention at one distinct point in time, neglecting that attention consists of different consecutive stages [13]. In addition, RT is influenced by non-attention-related processes like the speed of motor response, which may be generally delayed in MD [12]. The most frequently used paradigm, the Dot-Probe Task (DPT) [25], in which a probe appears in the location of either an emotional or a neutral stimulus, has been shown to have unacceptable psychometric properties [26–28]. The emotional Visual Search Task (VST) [29], an alternative paradigm measuring RTs to identify a target emotional stimulus among distractors, shows better reliability [30, 31].

A more direct approach to assess attention is registering overt visual attention by tracking eye movements [13]. In eye-tracking (ET) studies, free viewing paradigms like the Passive Viewing Task (PVT) [21] are used most frequently. Participants are presented with various emotional stimuli to look at without further instruction. This method is not influenced by (non-ocular) motor response and, more importantly, provides data of visual attention over a longer period of time, enabling to distinguish between the various components of visual attention (unlike RT based measures). As Högström et al. [32] elaborate, the vigilance-avoidance-hypothesis in research of AB in anxiety states that differentiating categorically between visual attention towards (vigilance) or away from (avoidance) salient stimuli might not grasp the attentional process accurately. Instead, there is evidence from eye-tracking research that individuals with anxiety show initial vigilance to disorder-related stimuli, followed by avoidance (see e.g. [32–34]). As both, initial orientation of attention as well as maintenance of attention can be assessed via the PVT, it allows to investigate if the vigilance-avoidance-hypothesis or similar more complex attentional mechanisms also apply to youth depression.

Additionally, psychometric properties of the PVT have been found acceptable in adults [27, 35] and in youth [36]. To our knowledge, only two studies have employed this paradigm to investigate bias in sustained attention in youth depression: one found AB away from sad faces in youth with MD [21], the other found AB towards sad faces in youth at risk for MD, moderated by genetical differences in HPA-axis reactivity [24].

### The Current Study

The study had two main aims. The *first aim* was to investigate whether negative AB are present in youth with MD and to characterise AB by (1) direction (attention towards or avoidance of negative emotional stimuli) and (2) specificity to emotions and to particular aspects of attention. To investigate specificity to emotion, we assessed biases for disorder-specific (sad) as well as non-specific (angry) faces. To investigate different aspects of attention, we used multiple age-adapted experimental tasks: the DPT as the most commonly used AB task, the VST, an RT-based measure guiding participants’ attention towards distinct emotions and the PVT, an ET based measure in which individuals freely gaze at the emotional stimuli.

We compared 9–14-year-olds^1^ with MD to two groups of non-depressed children and adolescents varying in their risk for depression: children of parents with a history of depression, known to have an increased risk for MD themselves (high-risk; HR), see e.g. [39] and children of parents with no history of any mental disorder with a low risk for depressive disorders (low risk; LR). The comparison of those three groups enabled us to pursue the study’s *second aim*: to determine whether AB are more pronounced in currently depressed youth compared to youth at elevated risk for depression. HR youth have been found to show AB for negative information, although their direction and specificity has not been established yet with evidence for AB towards sad [40, 41] or angry [42] stimuli, as well as for AB away from sad stimuli [43]. Negative AB in children and adolescents at risk for depression would indicate that AB might act as a cognitive vulnerability contributing to the development of depression, as suggested by theoretical models, e.g. [7, 8]. Even more pronounced AB in currently depressed children and adolescents however would indicate that these biases might be exacerbated as a consequence of depressive symptomatology.

No study to date has directly compared AB in depressed, HR and LR youth, however, studies on interpretation [44] and memory [45] biases have found negative biases to be present in both depressed as well as high-risk youth compared to low-risk youth but to be more pronounced in currently depressed youth than in high-risk groups.

With respect to our first aim we expected to find more negative AB in currently depressed youth compared to healthy youth (both high- and low-risk), in accordance with theoretical models [8] and previous research [18]. Regarding specificity, we expected AB for sad faces (i.e. mood-congruent depression-specific stimuli) rather than angry faces (i.e. threatening anxiety-specific stimuli) in both the MD and HR groups. Due to differences in direction of AB in previous studies, we did not make predictions on direction of AB, i.e., whether MD participants would look preferably towards negative stimuli or avoid them. With respect to our second aim, we expected negative AB to be present in youth at high risk for depression compared to youth at low risk for depression (according to [40–42, 46]) but to be more pronounced in the MD versus HR group, as found for interpretation and memory biases [44, 45].

## Material and Methods

Data on AB presented in this paper were collected as add-on to a study on cognitive biases in the offspring of depressed versus non-depressed parents [37]. Data from tasks comparing AB in MD, HR and LR youth are presented here. Data from interpretation bias tasks [38, 44] and tasks comparing AB in children and parents of HR and LR groups [36] are presented elsewhere. To ensure quality of our tasks and results, we determined psychometric properties of each task and parameter and only report those with acceptable reliabilities (PVT, VST). Additional tasks and results can be found in the supplement.^2^

### Participants

In total, 124 children and adolescents aged 9–14 years were included in data analysis.^3^ Participants formed three groups based on their current mental health state and risk of developing MD: n = 32 youth with MD (MD group), n = 49 mentally healthy youth at high familial risk for MD (HR group) and n = 43 mentally healthy youth with low risk for MD (LR group). Sample size was based on an a priori power analysis (α error probability = 0.05; power = 0.8; two-tailed) for the comparison of HR and LR groups (a smaller effect size was expected for this effect than for comparison with the MD group). Based on effect sizes (ES) from previous studies with mood induction procedures [40, 41], we expected an ES of at least d = 0.7, resulting in a required sample of n = 34 per group.

Participants with MD were recruited mainly at a University Hospital for Child and Adolescent Psychiatry in Germany, some by a cooperating licensed psychotherapist and some responded to public adverts. Data of the LR and HR groups was largely (87%) derived from a study evaluating the transgenerational transmission of cognitive biases [36, 38] in which children and adolescents participated with one of their parents. N = 29 HR participants were recruited from a previous study on a preventive intervention for this particular risk group [48], n = 12 of them had taken part in the programme by the time they were tested in the present study. Others, as well as children in the LR group, were recruited by local and online advertisements and by letters to randomly selected families with children in the corresponding age range provided by the local registry office.

Youth were included in the MD group if they currently met criteria for MD according to DSM-IV [49]. Of those 32 youths, n = 4 fulfilled criteria for a recurrent course of MD, n = 2 were in partial remission, n = 15 had at least one comorbid anxiety disorder and n = 3 were treated pharmacologically. The HR group comprised youth who did not meet criteria for any axis I disorder, current or past,^4^ but at least one of their parents fulfilled diagnostic criteria for MD (n = 47; of which n = 8 fulfilled diagnostic criteria at the time of participation) or dysthymia (n = 2) during the child’s lifetime. Youth were not included if their parents had a history of bipolar disorder, schizophrenia or substance abuse. Participants were included in the LR group if they did not meet criteria for any axis I disorder currently or in the past and none of their parents met criteria for any axis I disorder at any time. All participants had normal or corrected to normal vision. Exclusion criteria for participants in all three groups were an intelligence quotient (IQ) < 85,^5^ assessed with the CFT-20-R [50], a pervasive developmental disorder, attention deficit and hyperactivity disorder or a history of schizophrenia or bipolar disorder.

All procedures were approved by the ethics committee of the Medical Faculty of the LMU Munich. Written informed consent was obtained from all participants and parents after a comprehensive explanation of the experimental procedures. HR and LR youth who participated with their parents in the previous study on transgenerational transmission of cognitive biases received a reimbursement of €50 while MD participants who took part without their parents only in this study received €30.

### Psychopathology Assessment

Before inclusion, all participants underwent extensive diagnostic assessment. The K-DIPS (“Diagnostisches Interview bei psychischen Störungen im Kindes- und Jugendalter”) [51], a standardised, semi-structured psychiatric diagnostic interview for assessment of psychiatric diagnoses in youth, was administered to participants and one of their parents. The K-DIPS is a well-established German diagnostic measure based on diagnostic criteria of axis I disorders according to DSM-IV [50] with good interrater-reliabilities [52]. Interviews were conducted and evaluated by trained interviewers. Interrater-reliability was determined for 18% of HR and LR participants and revealed an accordance rate of 100% for lifetime diagnosis of depression (pre-defined criterion). Interviews from the MD group were not re-rated, but most MD participants were receiving treatment for a clinical diagnosis of depression that was confirmed with the diagnostic interview. Psychiatric diagnoses of LR and HR participants’ parents were assessed with the adult version of the interview, the Diagnostisches Interview bei psychischen Störungen (DIPS), [53], with good interrater-reliability [54]. Accordance rate for lifetime diagnosis of depression was 94% in our sample. In the HR group, the DIPS was administered only to the parent affected by depression; in the LR group the DIPS was administered at least to one parent and whenever possible to both parents.^6^ The German version of the Beck Depression Inventory-II [55], obtained from both parents for 83% of LR youth, was used to measure parents’ depressive symptoms. Scores differed significantly (t_64.5_ = 6.0, p < 0.001) between parents of HR (M = 9.9; SD = 8.5) and LR participants (M = 1.9; SD = 3.5).

For the assessment of the participants’ depressive symptoms the German version of the Children’s Depression Inventory “DIKJ” [56] was used and symptoms of anxiety were measured by the trait scale of the German version of the State Trait Anxiety Inventory for Children, STAIC [57]. For 123 of 124 included participants, a score for depressive symptoms could be determined and for 119 of 124 children an anxiety score was available. In our sample, reliability of both self-report measures was excellent (DIKJ: Cronbach’s α = 0.96; STAIC-T: Cronbach’s α = 0.92).

### Stimuli

Coloured photographs of children’s faces displaying sad, angry, happy and neutral expressions on black background served as stimuli in both tasks [58]. Half of the models were male, half female in each task. The stimulus set comprised photographs of 16 models with sad, angry and happy expressions in the VST and 24 models with sad, angry, happy and neutral expressions in the PVT.

### Visual Search Task (VST)

An emotional VST, adapted from [29] was used to assess AB in an RT-based task with explicit instructions to attend to specific emotions. The task was administered during ET, but due to poor reliability of the ET indices, ET results are reported in the supplement.

**Fig. 1:**
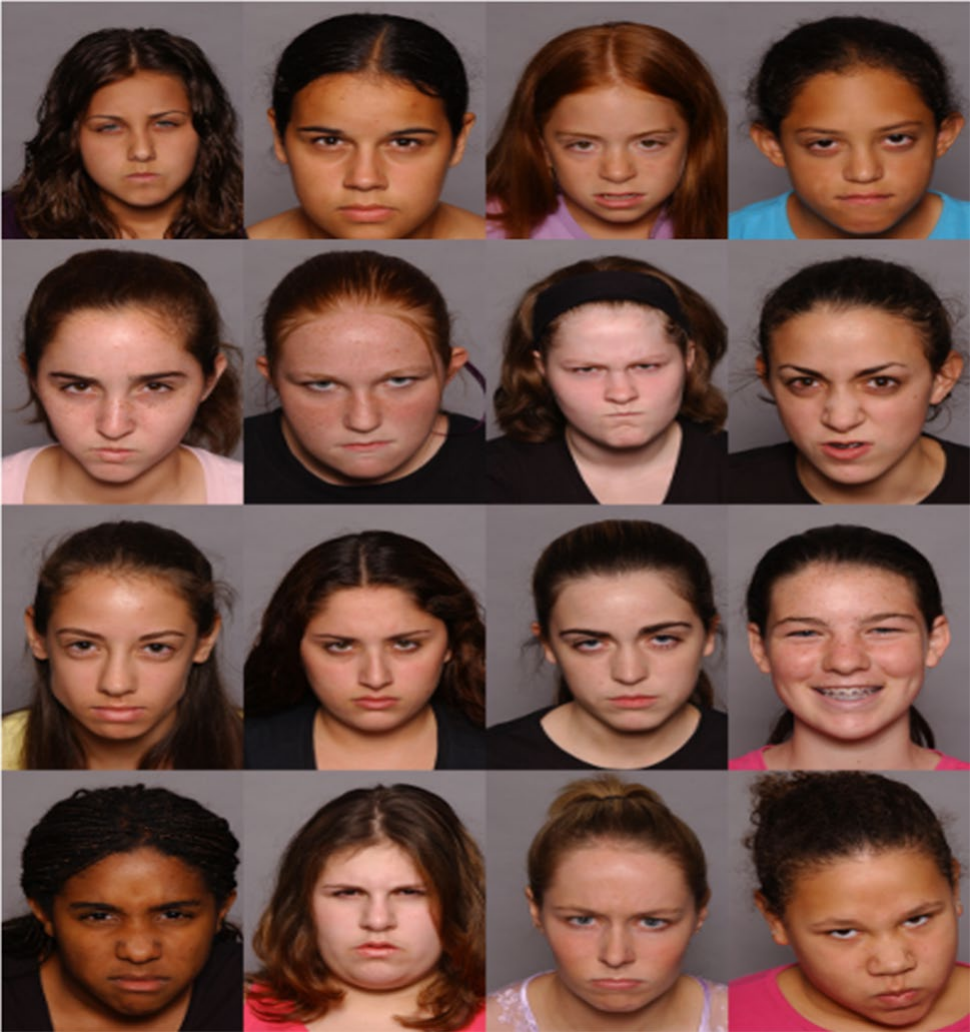
Example of an emotional trial of the Visual Search Task (VST; [29])

Each trial started with a drift correction (small white circle in the screen’s centre). Upon fixation of the circle, the trial was initiated. A fixation cross was then presented for 500 ms in the screen’s centre. Subsequently, the stimuli were presented in a 4 × 4 grid containing 15 distractors and one target (see Fig. 1). Each stimulus display contained all 16 models randomly allocated to the 16 grid positions. The participants’ task was to identify the face showing a certain emotion (i.e., the target) as quickly as possible and click on it with the mouse. Time to identify the target was unlimited. The experiment consisted of four blocks: Two blocks with happy targets and negative (either sad or angry) distractors and two with negative targets and happy distractors. Each block comprised 32 trials with the target appearing twice in each position and being twice each model. Blocks as well as trials within the blocks appeared in random order. Before each block, participants completed three practice trials.

#### Data Processing and Outcome Variables

Trials with incorrect responses or RTs shorter than 200 ms or longer than 2 SDs above each participant’s mean were excluded, in line with previous studies, e.g. [24]. Participants with poor accuracy (a correct and valid trial rate of 2 SDs below mean rate) were identified as outliers in terms of accuracy and excluded (n = 1 MD; n = 2 HR; n = 1 LR). In the remaining sample of 120 children, on average 119.6 correct and valid trials per participant (SD = 1.9; 93% of 128 trials; no group differences, Fs < 1, ps > 0.1) were available for analysis of the behavioural data.

A bias score (AB_VST_) was calculated by subtracting the mean RT in blocks with negative targets and positive distractors from the mean RT in blocks with positive targets and negative distractors [29, 59]. Hence, positive bias scores indicate more distraction by negative information (i.e. negative AB) and negative bias scores indicate more distraction by positive information (i.e. positive AB). AB_VST_ scores for sad and angry faces were computed separately.

#### Reliability

To investigate internal consistency for the VST, split-half reliability was assessed by correlating bias scores based on odd versus even trials [60]. Reliability of the behavioural bias score was acceptable for both emotions (sad: r = 0.83, p < 0.01, Spearman-Brown corrected: 0.91; angry: r = 75, p < 0.01, Spearman–Brown corrected: 0.86).

### Passive Viewing Task (PVT)

**Fig. 2:**
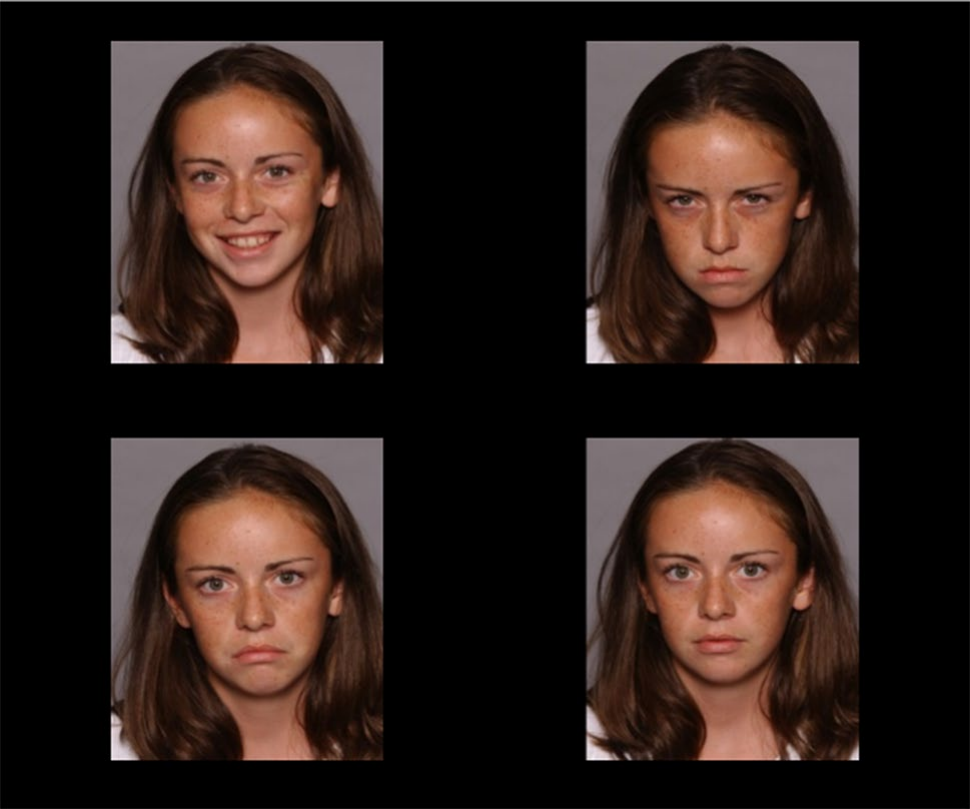
Example stimulus display from the Passive Viewing Task (PVT; [20])

As a purely ET-based measure of AB, enabling participants to freely gaze at the emotional stimuli, a PVT was administered [21]. Each trial began with a drift correction (see above). A fixation cross followed for 1000 ms. Then the 2 × 2 stimulus array was presented for 15000 ms. The task consisted of 16 emotional trials, corresponding to the minimum trial number suggested for ET research [61] and eight neutral trials (not analysed), presented in random order. In the emotional trials, each array consisted of four photographs of the same model displaying a sad, angry, happy and neutral facial expression (see Fig. 2). The position of each emotional facial expression was randomly assigned to one of the quadrants with each emotion being presented four times in each quadrant. The neutral trials comprised four photographs of the same model with a neutral facial expression. Stimuli were sized approximately 9.5 cm × 7.5 cm and were presented with a distance of approximately 6.5 cm horizontally and 1 cm vertically between. Participants were instructed to fixate the white circle and fixation cross and then freely view the stimuli, required only to keep their attention focussed on the screen.

#### Eye-Tracker

Eye movements were registered with an EyeLink 1000 Plus Desktop mounted eye-tracker (SR Research). Participants were seated in front of a 15-inch monitor (1024 × 768 pixel resolution) on which the experiments were presented using Experiment Builder 1.10 (SR Research Ltd., 2013). Viewing was binocular while eye movements were registered from the dominant eye via infrared video-based tracking technology with a sampling rate of 1000 Hz. A forehead and chin rest minimized head movements and kept the viewing distance constant at 65 cm. Lighting of the room was kept constant for all participants. Before each task, a 9-point calibration and validation procedure was conducted and calibration was accepted if the average error was less than 0.5° of visual angle and the maximum error was less than 1° of visual angle.

Eye movement events were detected using a velocity and acceleration-based saccade detection method with saccades defined, in line with other studies [e.g., 28, 62], as events with a velocity above the threshold of 30°/s or an acceleration above the threshold of 8000°/s2. Gaze positions that were stable within 1° of visual angle for at least 100 ms were defined as fixations (in line with other studies [e.g., 63].

To ensure data quality, trials with a total dwell time of less than 75% of the presentation time were excluded due to excessive blinks or missing data [62] and ET data of each participant was inspected for systematic calibration errors. Subsequently, participants with less than 70% valid trials [64] and systematic calibration errors were excluded from the analyses of the eye-tracking data (n = 3 MD, n = 3 HR and n = 1 LR).^7^

#### Data Processing and Outcome Variables

After exclusion, a remaining sample of 115 children with on average 15.3 trials (SD = 1.1; 95.6% of 16 trials) per participant were available. This did not differ between groups (F ≤ 1.8; ps > 0.1).

As an indicator of initial orientation of attention, percentages of location of the first fixation on each emotion were assessed. As this parameter showed poor reliability, results are reported only in Supplement 2. As indicator of maintenance of attention, percentages of dwell time (defined as summation of all fixations) on each of the four emotions were assessed. For appraising the course of attention over time, trials were divided into five time windows (each 3000 ms).

#### Reliability

To assess reliability of the PVT, Cronbach’s alpha was calculated for percentage of dwell time on all four emotions (as for this task no AB scores were computed). Reliabilities for percentage of dwell time ranged from acceptable to good (angry: α = 0.69, happy: α = 0.86, neutral: α = 0.83, sad: α = 0.86).

### Procedure

The course of the experimental session corresponds to [38]. Tasks were presented in random order and a mood induction procedure was administered twice during the experimental session. Participants watched a 2 min scene from the movie The Lion King [64] that had successfully induced unpleasant mood in children and adolescents before [65]. In our study, participants in both healthy groups reported a significantly worse mood, assessed using the valence dimension of the 9-point Self-Assessment Mannequin scale [66] after watching the movie scene compared to baseline (Fs ≤ 43.22; ps < 0.001). Mood did not change significantly, however, in the MD group (F2.4 = 1.15; p = 0.33). Details are presented in Supplement 5.

### Data Analysis

Statistical data analysis was conducted with SPSS25. Significance level was set to p = 0.05 (two-tailed).

For the VST, one-way analyses of variance (ANOVAs) were conducted to assess group differences in AB_VST_ scores. To assess relationships between psychopathology and AB, correlations were calculated of AB_VST_ score with depression and anxiety scores.

For the PVT, a repeated-measures ANOVA with within-subject factors Time Window (5: time windows 1–5) and Emotion (4: angry, happy, neutral, sad) and the between-subjects factor Group (3: MD, HR, LR) were performed for percentages of dwell time. Degrees of freedom were adjusted via Greenhouse–Geisser correction when necessary. As this study’s main focus was a group comparison, only significant effects involving the factor Group were followed up using post-hoc ANOVAs and t-tests. To assess relationships with psychopathology, correlations of dwell time on each emotion (averaged across time windows) with depression and anxiety symptoms were computed.

All analyses were repeated excluding participants with psychopharmacological treatment (n = 3), as this has been found to reduce negative cognitive biases [67] and enhance attention toward positive stimuli [68]. The pattern of results in our sample remained the same, so findings are reported based on the whole sample. We did not include age as a covariate in our analyses as this is not appropriate when the between-groups factor (group) and covariate (age) are non-independent [69]. However, we performed additional analyses to investigate if age may have accounted for our results (see below).

## Results

### Sample Characteristics

Sample characteristics are presented in Table 1. Groups did not differ in gender ratio or IQ but in age: participants in the MD group were significantly older than participants of the HR and LR groups. As expected, the groups also differed in psychopathology: the MD group reported higher depression and anxiety symptoms than the healthy groups, which did not differ from each other, indicating that the HR group was as psychiatrically healthy as the LR group.

**Table 1:** Demographic and clinical characteristics

	MD	HR	LR	ANOVA	Post hoc tests	
	*n* = 32	*n* = 49	*n* = 43			
Gender m/f	6/26	19/30	18/25	*χ*^*2*^ = 4.9	n.s	
Age; M (SD)	13.4 (1.4)	11.8 (1.7)	12.1 (1.7)	*F*_2,121_ = 9.8	*p* < .001	MD > HR = LR
IQ; M (SD)	105.2 (13.6)	108.8 (11.5)	111.8 (10.2)	*F*_2,121_ = 2.9	n.s	
Depressive symptoms; M (SD)	31.5 (8.9)	7.9 (5.7)	6.6 (5.2)	*F*_2,120_ = 163.5	*p* < .001	MD > HR = LR
Anxiety; M (SD)	45.1 (8.8)	30.0 (6.4)	28.1 (6.2)	*F*_2,116_ = 56.6	*p* < .001	MD > HR = LR

Depressive symptoms were assessed with DIKJ (raw values presented), anxiety with STAIC-T. All post-hoc tests were significant (*p* < .001)

*MD* major depression group, *HR* high-risk group, *LR* low-risk group, *M* Mean, *SD* Standard Deviation

### Visual Search Task

One-way ANOVAs on AB_VST_ scores revealed no significant differences between MD, HR and LR youth (Fs < 1; ps > 0.1) regarding sad or angry faces. The AB_VST_ for sad faces was significantly < 0 in the LR group (t_41_ = 2.5, p = 0.016), indicating a positive AB for sad faces (i.e. away from sad faces/towards happy faces), but bias scores for sad faces in the other groups as well as for angry faces were not significantly different from 0 (ts ≤ 1.6, ps > 0.1). Bias scores are presented in Table 2. The correlational analyses revealed no relationship between AB_VST_ scores for sad or angry faces with depressive or anxiety symptoms (r ≤ 0.17; p ≥ 0.072).^8^

**Table 2:** AB scores from the Visual Search Task

AB_VST_	MD	HR	LR
	*n* = 31	*n* = 47	*n* = 42
AB_VST_ for sad faces M (SD)	− 47.0 (1040.1)	− 259.2 (1098.6)	− 323.8 (832.6)
AB_VST_ for angry faces M (SD)	− 11.0 (882.2)	227.4 (1227.9)	89.2 (884.6)

*AB*_*VST*_ attention bias score from the Visual Search Task, *MD* major depression group, *HR* high-risk group, *LR* low-risk group, *M* Mean, *SD* Standard Deviation

### Passive Viewing Task

**Fig. 3:**
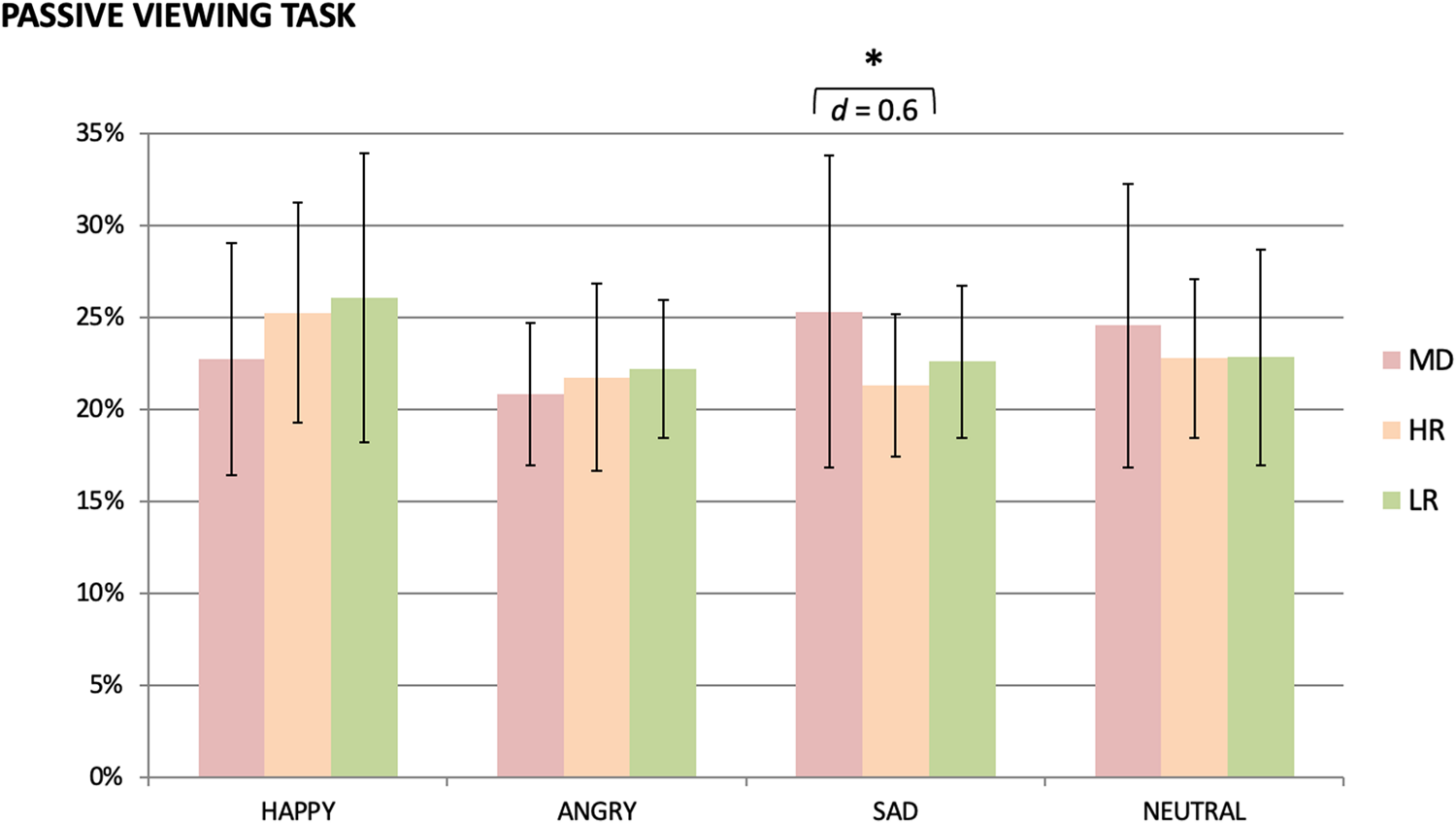
Percentage of dwell time on emotional faces among groups. *MD* major depression group, *HR* high-risk group, *LR* low-risk group. * MD and LR group differ significantly in their dwell times on sad faces. Error bars represent standard deviations

The TimeWindow × Emotion × Group ANOVA revealed a significant main effect of TimeWindow (F_1.9,207.3_ = 24.3, p < 0.001, η_p_^2^ = 0.18) and Emotion (F_2.6,295.1_ = 4.5, p = 0.006, η_p_^2^ = 0.04). Significant TimeWindow × Emotion (F_9.6,1076.0_ = 1.9, p = 0.040, η_p_^2^ = 0.02) and Emotion × Group (F_5.3,295.1_ = 2.5, p = 0.027, η_p_^2^ = 0.04) interactions emerged. All other main effects and interactions were non-significant (Fs ≤ 2.3; ps ≥ 0.1). To follow up the Emotion × Group interaction, ANOVAs with the factor Group were calculated separately for each emotion on percentage of dwell time averaged across time windows: a significant effect emerged of Group for sad faces (F_2,112_ = 4.7, p = 0.011, η_p_^2^ = 0.08), but no other emotion (Fs ≤ 2.5, ps ≥ 0.08). Post-hoc t-tests revealed a significant difference in percentage of dwell time on sad faces between the MD and HR groups (t_36.2_ = 2.3, p = 0.027, d = 0.6) with the MD group dwelling more on sad faces than the HR group. Differences between MD vs. LR groups (t_33.4_ = 1.5, p = 0.145, d = 0.4) and between HR and LR groups (t_85_ = 1.7, p = 0.090, d = 0.4) were non-significant, with a trend for HR participants dwelling less on sad faces than LR participants. Percentages of dwell time are presented in Fig. 3. Furthermore, positive correlations of dwell time on sad faces with depressive (r = 0.29, p = 0.002) and anxiety symptoms (r = 0.38, p < 0.001) and a small negative correlation between dwell time on happy faces and depressive symptoms (r = -0.21 p = 0.023) were found among all participants.^9^

### Additional Analyses

As groups differed in age, we performed additional analyses to investigate if this may have accounted for the group differences in dwell times on sad faces. Pearson’s correlations between age and dwell time on sad faces were computed separately within each group (to rule out that correlations were artifacts of group differences): no significant correlations emerged (rs ≤ 0.23, ps > 0.1).

## Discussion

The overarching goal of the present study was to investigate the nature of negative AB in depressed youth. With two different experimental approaches, a passive viewing ET paradigm and an RT-based task externally guiding attention, we evaluated AB in three groups of 9–14-year-olds: currently depressed youth (MD group), mentally healthy youth with a high familial risk for depression (HR group) and with a low familial risk for depression (LR group). While the behavioural paradigm revealed no evidence of AB in any group, the eye-tracking paradigm revealed a more negative bias towards disorder-specific stimuli in depressed compared to high-risk youth in maintenance of attention. This measure correlated with depressive symptoms across groups.

Our *first aim* was to characterise AB in depressed youth regarding presence, direction, specificity to disorder-related information and specificity to particular attentional components. Our *second aim* was to determine to which extent AB are more pronounced in currently depressed youth compared to at-risk youth.

We found a significant group difference in dwell time on sad faces in the PVT between youth with MD and at-risk youth: as expected, youth with MD dwelled longer on sad faces compared to youth at risk for depression. Contrary to our expectations, the LR group’s dwell time on sad faces was in between: they dwelled less on sad faces than the MD group but more than HR youth (although these differences were non-significant). No differences were found for the other emotions. These results partially support our hypotheses: they suggest that youth with MD might show more negative AB in maintenance of attention on disorder-specific negative stimuli in comparison to healthy children. However, according to our results, this would appear to be the case only for healthy children with a familial risk of depression.

Youth with MD preferentially attending to disorder-specific negative information is in line with several studies [19, 20], nevertheless, the only study that used a similar passive viewing ET paradigm [21] found that youth with MD looked less at sad faces, i.e. avoided them. These contradicting results might be explained by the age of the investigated samples: while the mean age of the depressed groups in the studies by Hankin et al. [19], Sylvester et al. [20] and ours was above 13 years, Harrison and Gibb [21] examined a considerably younger sample with a mean age of 11.2 years which might have captured youth before a “maladaptive shift” in their emotion regulation starting approximately from age 12 [70, 71]. Harrison and Gibb [21] argue that children may use avoidance of sad faces to regulate negative emotion and that this ability may diminish with age. Thus, it would be lost in older adolescents and adults with depression, leaving them to preferentially attend to negative information. A similar idea of a change in attention bias with age is found in research on AB for threatening stimuli in youth with anxiety disorders: as Dudeney et al. [33] showed in their meta-analysis, differences in AB to threat between children with anxiety diagnosis and control groups are found to increase with age. This change might be due to a general tendency in children to be more immediately reactive to external stimuli, which would normally decrease over time as their ability to inhibit attention and information processing develops. Children and adolescents who develop an anxiety disorder might instead maintain this heightened attention towards threat [34]. If this reactivity would not only apply to threatening stimuli, but also to other salient stimuli, the same development might potentially occur regarding AB towards sad stimuli.

Although not statistically significant, our HR group showed a slightly *less* pronounced negative AB compared to the LR group. This pattern of results was somewhat surprising as AB in high-risk youth are proposed to act as a risk factor for depression [46] and to be exacerbated during an episode of depression (as memory and interpretation biases [44, 45]) and are therefore expected to have the same direction as AB in currently depressed youth. As attentional deployment is an effective emotion regulation strategy (see e.g. [9, 72]), AB away from negative information might act as a protective mechanism against development of depression. Our sample of children of depressed parents tended to show AB away from sad faces, as described for mentally healthy youth [19, 73]. It is conceivable, albeit rather speculative, that they might have acquired this mechanism as a protective response to their parents’ disorder. This might add to their resilience, helping to explain why they are still psychiatrically healthy despite their heightened risk for MD.^10^ Maybe at-risk youth who do not apply this protective mechanism would be more vulnerable to depression and may have already developed a depressive disorder [74]. In line with this assumption, participants in our MD group did not show this protective mechanism—either because their ability to avoid negative information was already less pronounced before, adding to their vulnerability, or because their ability to regulate their emotions this way diminished as a consequence of depressive symptomatology [75]. Due to the cross-sectional design of our study, we cannot make conclusions about time course or causality, i.e. whether the more negative AB in the MD group compared to the HR group is a consequence of depression, present before disorder onset, or if the use of avoidance of negative stimuli as an emotion regulation strategy decreases with age as suggested by Harrison and Gibb [21]. However, these deliberations are highly speculative since differences between HR vs. LR and MD vs. LR did not reach significance, the difference between HR and MD groups would not have been significant if a correction for multiple testing had been applied and effect sizes for differences between groups were smaller than expected.

Other indices of attention we investigated yielded either non-interpretable results due to poor reliability (e.g., location of the first fixation in the PVT as index of initial attention allocation; see Supplement 2) or did not reveal any group differences despite acceptable reliability (e.g., the behavioural AB_VST_ parameter). In the VST, the LR group was found to show a small positive AB regarding sad faces, but no significant biases emerged for HR or MD groups as well as no group differences. Since the VST was originally designed to modify AB and measure within-subject differences in a Cognitive Bias Modification Training [29, 76], it might not be sensitive to between-group differences. Moreover, the VST is based on distinct instructions which emotion to attend to, i.e. attention is guided externally, whereas in the PVT, participants can deliberately choose where to turn their attention. In our study, evidence for AB in depressed youth was found only with the PVT, not the VST, suggesting that depressed and high-risk youth differ only in an internally controlled and at least to some extent conscious aspect of attention. This is in line with Teachman et al. [77] who found evidence for a more conscious, intentional bias in attention. Importantly, the measure revealing group differences—percentage of dwell time on sad faces in the PVT—not only showed good reliability but was also related to depressive symptoms across groups, suggesting it is a valid indicator of “depressive” processing.^11^ Of note, dwell time as a measure of AB cannot be easily attributed a certain component of attention. Longer dwell times, i.e., longer maintenance of attention on certain stimuli, may result from participants more frequently looking at them, having difficulties disengaging their attention from them, or deliberately maintaining their attention on them. Future studies may try to tease apart those mechanisms in order to identify which is or are responsible for our results.

### Strengths

To our knowledge, this is the first study directly comparing AB in currently depressed, high-and low-risk youth. It contributes to a more detailed understanding of AB in youth depression and their role in development and maintenance of MD. Another strength is our use of a standardised, semi-structured diagnostic interview with all participants, conducted by trained personnel to ensure diagnostic accuracy. Moreover, parental psychopathology was also assessed via a psychiatric interview in the HR and LR groups rather than relying on self-report measures. In addition, we used multiple measures of AB, assessing attention in deliberate vs. externally guided circumstances. We evaluated reliabilities of our measures, a practice often omitted in AB research [36], and only interpreted the results of those measures with at least acceptable reliability. This led to differing results, contributing to a more nuanced understanding of AB in youth depression.

### Limitations

With most MD participants recruited at a Department of Child and Adolescent Psychiatry or through outpatient psychotherapists, most had received psychotherapy at the time they participated. Unfortunately, we did not systematically assess treatment type or duration. As psychotherapy is expected to reduce AB (e.g. in anxiety disorders; see e.g. [78–80]) effect sizes might be underestimated in our study. Furthermore, we recruited the HR group partly from a study evaluating a prevention program for at-risk youth [49] some had already participated in. This Cognitive Behavioural Therapy based group intervention conveyed distraction as a coping strategy, among others, which might have influenced participants’ volitional attention maintenance and added to our HR group’s resilience, rendering them not entirely representative for children of depressed parents. Another limitation results from comorbid anxiety disorders in nearly half of the participants in the MD group, known to be associated with AB, as well [80]. However, anxious youth are found to show AB for threatening stimuli [80–82] while depressed individuals are commonly found to show AB for sad stimuli [13]. Since we found AB for sad faces only, it is more likely that the AB we captured were related to participants’ depression rather than the comorbid anxiety disorders. In addition, our three groups differed significantly in age with the MD group being the oldest. As the incidence of MD increases with puberty [83] this does not come as a surprise. However, since the detected AB did not correlate with age, it is unlikely that this difference accounted for our results. Furthermore, it has to be emphasized that although our sample size is larger than previous studies’ (e.g., [21]: n = 19 in the MD group; [23]: n = 19 in the MD group; [73]: n = 10 in the high-risk group), the sample size was only powered to detect medium ES and may have prevented us from finding smaller effects. At the same time, it is possible that the significant effect we found is not a real group difference but a false positive result since we did not correct for multiple testing (in line with, e.g. [21]). Replication in larger samples of children and adolescents is therefore crucial to rule out that our result is a chance finding and to be able to draw firmer conclusions.

Finally, even though we determined and reported reliabilities of our measures and the correlation of the PVT maintenance score with depressive symptoms indicates that it could be a valid indicator of depressive processing, we cannot provide a full description of the psychometric properties of our tasks. The appropriateness of measures that have often been used to assess AB in the past, like the DPT, has repeatedly been called into question [26–28], while at the same time no alternative measure has yet become the widely accepted norm. Therefore, future studies should further examine reliability and validity of tasks assessing ABs, refine existing tasks and develop new ones that can measure AB with less error. As this should reduce the variability of results, it would truly move the field forward.

### Summary

In summary, our study provides preliminary evidence for a more negative AB in depressed youth compared to healthy youth at risk for depression. Our sample of youth with MD preferentially attended to sad faces, i.e., their AB was directed towards negative information. This AB was found only regarding sad, not angry faces, suggesting AB in youth depression may be specific to disorder-related negative emotional information. In addition, the more negative AB in depressed youth was found only for maintenance of attention in a task where attention was allocated deliberately, suggesting this AB might be specific to an internally controlled and at least to some extent conscious aspect of attention. Our results suggest that depressed and at-risk youth might show AB in opposite directions with AB towards negative information in depressed youth and AB away from negative information in at-risk youth, potentially acting as a protective mechanism in this group. However, this interpretation remains highly speculative and replication in larger samples is essential. Longitudinal research is necessary to address the temporal course of AB in youth, i.e. if youth with AB away from negative information are less likely to develop a depressive disorder than those with negative AB towards or if negative AB arises with depressive symptomatology. This will help shed more light on the role negative AB play in the development and maintenance of depressive symptoms. We think that despite the speculative nature of our interpretations they provide an interesting incentive for the elaboration of theoretical frameworks and starting points for future investigations.

## Supplementary Information

Below is the link to the electronic supplementary material.

Supplementary file1 (DOCX 124 kb)

## Footnotes

1 As the present study was planned as add-on to a study on transgenerational transmission of cognitive biases 37, 38, it focused on the same age range. Children younger than 9 years were not included due to concerns about their ability to understand and perform the tasks. Adolescents above 14 years were not included since the incidence of MD in children of depressed parents increases substantially after that age, e.g. 39, and investigating older mentally healthy children of depressed parents might result in examining a particularly resilient, thus non-representative sample.

2 In addition to PVT, VST and DPT, we aimed to assess AB with a computerized version of the Scrambled Sentences task (SST, 47) via ET in addition to interpretation bias (IB; see 44 for results on IB). Due to poor reliability, DPT and SST are included in the supplement.

3 126 children and adolescents were tested; two were excluded due to poor compliance.

4 One HR participant had met criteria for enuresis in the past, but did not report any other symptoms of any other mental disorder, so she was included nonetheless.

5 Two MD group participants scored just below 85. The substandard IQ did not correspond to the clinical impression and was most likely due to a lack of compliance and inability to concentrate in the context of their MD. Therefore, they were still included.

6 This was possible for 79% of LR participants. For the other 21% the second parent was not available for the interview, for example because participants grew up in a single parent household with little or no contact to the second parent. We still included those children and adolescents as excluding them would have decreased representativeness of the sample.

7 PVT: Data of 1 MD and 1 HR participant were unavailable due to technical difficulties.

8 As reaction times were not normally distributed, we repeated our analyses with bias scores calculated from median instead of mean reaction times as well as bias scores based on square root transformed reaction times. The pattern of results did not change.

9 As groups differed in psychopathology scores and dwell time, correlational analyses were repeated within each group: correlations between dwell time on sad faces and depressive symptoms as well as anxiety scores were non-significant (rs ≤ .28, ps ≥ .083).

10 Note that in our HR group, only children of depressed parents who did *not* meet criteria for any axis I disorder (current or past) were included, i.e. only the still mentally healthy. Therefore, this sample of HR children is not representative of unselected children of depressed parents, expected to show more pronounced psychopathology on average.

11 It has to be noted that dwell time on sad faces was not specifically related to depressive symptoms but also to anxiety scores.
