# Supplementary material for: Biased Maintenance of Attention on Sad Faces in Clinically Depressed Youth: An Eye-Tracking Study
Source: Child Psychiatry Hum Dev. 2021 Sep 2;54(1):189–201. doi: 10.1007/s10578-021-01229-z (PMC9867681; doi:10.1007/s10578-021-01229-z)
Supplement: Supplementary file 1 — Supplementary file1 (DOCX 124 kb) [file 10578_2021_1229_MOESM1_ESM.docx]

# Supplement 1: Visual Search Task eye-tracking results

An emotional Visual Search Task (VST; adapted from [1]) was administered during Eye-Tracking (ET) to assess overt visual attention to the different emotional stimuli during the task in addition to the reaction time (RT) based measure of attention bias (AB). RT results are reported in the manuscript, while ET results are reported here.

## Task procedure. The task procedure is described in the manuscript.

## ET data processing and outcome variables. In addition to excluding trials with incorrect responses or RTs shorter than 200ms or longer than 2 SDs above each participant’s mean, (trials with poor data quality (see data processing of the Passive Viewing Task ET data in the manuscript for criteria) and participants with insufficient trials available (n=1 MD) were excluded. 118.1 correct and valid trials per participant were available for the ET analysis in VST (SD = 4.0; 92.3% of 128 trials). This did not differ between groups (F ≤ 1.8; ps > .1).

An ET based AB score (AB_VST-ET_) was calculated by subtracting the mean percentage of dwell time on positive distractors from the mean percentage of dwell time on negative distractors. Hence, positive values indicate more attention towards positive information (i.e. a positive bias) and negative values indicate more attention towards negative information (i.e. a negative bias). AB_VST-ET_ scores for sad and angry faces were computed separately.

## Reliability. Split-half reliability was assessed by correlating bias scores based on odd versus even trials (see e.g. [2]). The reliabilities of the AB_VST-ET_ scores were poor for both emotions (angry: r = .13, p = .378, Spearman-Brown corrected: .23; sad: r = .37 p = .011, Spearman-Brown corrected: .54).

## Data analysis. One-way analyses of variance (ANOVAs) were conducted to assess group differences in AB_VST-ET_ scores. To assess relationships between psychopathology and AB, correlations were calculated between AB_VST-ET_ scores and depression as well as anxiety scores.

## We found results on reaction times in the VST were inconsistent with a gaussian distribution using Kolmogorov-Smirnov (: ≤ .15, ps ≤ .001) and Shapiro-Wilk tests (≤.96, ps ≤ .001). The histogram showed them to be mildly right-skewed instead.

## We therefore determined the median RT instead of the mean RT and proceeded to calculate bias scores using the median of reaction times instead of their mean.

## Results. The one-way ANOVA on AB_VST-ET_ revealed no significant group differences between MD, HR and LR groups (Fs < 1, ps > .1) for either sad or for angry faces. No correlations between dwell time and depression or anxiety scores emerged (|rs| ≤ .18; ps > .05).

One-way ANOVAs on AB_VST_ scores (calculated with median) revealed no significant differences between MD, HR and LR youth (Fs < 1.2; ps > .3) regarding sad or angry faces. The correlational analyses revealed no relationship between AB_VST_ scores for sad or angry faces with depressive or anxiety symptoms (r ≤ .17; p ≥ .07).

In addition, we performed a transformation of the mean RTs using the square root to achieve gaussian distribution. With the transformed data, we then calculated AB bias scores in the same manner as in the original manuscript. One-way ANOVAs on AB_VST_ scores revealed no significant differences between MD, HR and LR youth (Fs < .71; ps > .49) regarding sad or angry faces.

**Supplementary table 1.** Eye-tracking AB scores for negative emotional faces (sad; angry) among all 3 groups in VST.

|  | **MD** | **HR** | **LR** |
| --- | --- | --- | --- |
|  | *n* = 30 | *n* = 47 | *n* = 42 |
| AB_VST-ET_ for sad faces; *M (SD)* | -1.5% (6.7%) | -1.8% (6.1%) | -1.3% (5.4%) |
| AB_VST-ET_ for angry faces; *M (SD)* | -1.2% (4.8%) | 0.4% (6.2%) | -0.6% (5.2%) |

Note: AB_VST-ET_ = eye-tracking attention bias score from the Visual Search Task; MD = major depression group; HR = high-risk group; LR = low-risk group; M = Mean; SD = Standard Deviation.

## Interpretation of results. The results show no evidence for group differences in AB between the groups. However, due to the poor reliability of the task the interpretation is limited. As reaction times were not normally distributed, we repeated our analyses with bias scores calculated from median instead of mean reaction times as well as bias scores based on square root transformed reaction times. The pattern of results did not change.

## References

1. De Voogd, E, Wiers, R, Prins, P, Salemink, E (2014). Visual search attentional bias modification reduced social phobia in adolescents. J Behav Ther Exp Psychiatr 45(2):252-259.
2. Van Bockstaele, B, Salemink, E, Bögels, SM, Wiers, RW (2017). Limited generalisation of changes in attentional bias following attentional bias modification with the visual probe task. Cogn Emot 31(2):369-376.

# Supplement 2: Passive Viewing Task

## Initial orientation of attention. A Passive Viewing task (PVT) as a purely ET based measure of attention bias (AB) that allows investigation of different components of attention was administered [1]. Percentages of dwell time on each of the four different emotions were assessed as an indicator of maintenance of attention while percentage of location of the first fixation after trial onset were assessed as an indicator of initial orientation of attention. However, as the latter parameter showed poor reliability, results are reported only in this supplement.

## Task procedure. The task procedure is described in the manuscript.

##

## Data Processing. Data processing is described in the manuscript.

## Reliability. To assess reliability, split-half reliability was calculated for percentage of location of the first fixation on all four emotions: it ranged from r = -.09 (Spearman-Brown corrected: .16) to -.03 (Spearman-Brown corrected: .06); all p > .32.

## Data analysis. In order to compare first fixations, ANOVAs with within-subject factor EMOTION (4: angry, happy, neutral, sad) and between-subjects factor GROUP (3: MD, HR, LR) were calculated. Degrees of freedom were adjusted via the Greenhouse-Geisser correction when necessary. As the main focus of the study was to compare MD, HR and LR groups, only significant effects involving the factor GROUP were followed up using post-hoc ANOVAs and t-tests. To assess relationships with psychopathology, correlations between percentage of first fixation on each of the emotions and depression and anxiety symptoms were computed.

## Results. Descriptive data for ET indices is presented in Supplementary table 2. The Emotion × Group ANOVA revealed no significant effects (Fs ≤ 1.6, ps > .1). No significant correlations between percentage of first fixations on neither sad faces nor any of the other emotions (|rs|≤ .04, ps ≥ .59) and depressive symptoms as well as anxiety scores (|rs|≤ .06, ps ≥ .1) were found.

**Supplementary table 2.** Percentages of First Fixations on each of the 4 emotional faces among all 3 groups.

|  | **MD** | **HR** | **LR** |
| --- | --- | --- | --- |
| **Percentages of First Fixations (%)** | *n* = 28 | *n* = 45 | *n* = 42 |
| Sad faces *M (SD)* | 22.9 (9.5) | 25.5 (11.8) | 23.5 (9.4) |
| Angry faces *M (SD)* | 26.3 (10.1) | 23.3 (10.8) | 21.7 (10.9) |
| Happy faces *M (SD)* | 25.1 (11.3) | 25.6 (10.7) | 28.7 (10.1) |
| Neutral faces *M (SD)* | 25.5 (11.8) | 25.7 (12.1) | 26.1 (12.7) |

Note: MD = major depression group; HR = high-risk group; LR = low-risk group; M = Mean; SD = Standard Deviation.

## Interpretation of results. These results show no evidence for group differences in initial orientation of attention between the groups. However, due to the poor reliability of the parameter the interpretation is limited. The good reliability of the maintenance of attention index and poor reliability of the initial orientation index is in line with e.g. [2] who found that the reliability of ET measures is higher for indices measured over longer (versus shorter) periods of time.

## References

1. Harrison, A. J., & Gibb, B. E. (2015). Attentional Biases in Currently Depressed Children: An Eye-Tracking Study of Biases in Sustained Attention to Emotional Stimuli. J Clin Child Adolesc Psychol 44(6):1008-1014.
2. Lazarov, A, Ben-Zion, Z, Shamai, D, Pine, DS, Bar-Haim, Y (2018). Free viewing of sad and happy faces in depression: A potential target for attention bias modification. J Affect Disord 238:94-100.

# Supplement 3: Dot Probe Task

A modified version of the Dot Probe Task (DPT; [1, 2, 3]) was used to assess ABs for sad as well as angry faces via reaction times (RT). Due to concerns about the task’s reliability that have been raised previously [4, 5] and the poor reliability we found in our previous study on AB in depressed parents and their offspring (|*r*| < .1; [6]), we decided not to include this task in the manuscript.

## Stimuli. Stimuli were coloured photographs of faces displaying angry, sad, and neutral emotional expressions. Stimuli were age-matched, i.e. children viewed pictures of child models from the NIMH Child Emotional Faces Picture Set (NIMH-ChEFS; [7]). Five female and five male models were selected and images (13.5 × 10.5cm) were presented on a black background.

## Task Procedure. The trial procedure is depicted in Supplementary Figure 1. Participants were seated in front of a 24-in. computer screen at a viewing distance of approximately 65cm. The experiment was presented using E-Prime 2.0 (Psychology Software Tools, 2013). Each trial started with a fixation cross that was presented for 1000ms at the centre of the screen. Then the face stimuli were presented for 500ms. Two pictures of the same actor were presented side-by-side: either an emotional (sad or angry) expression paired with a neutral expression (emotional trials) or two neutral expressions (neutral trials). The faces were followed by the probe which appeared in the location of one of the faces for 100ms, a timespan chosen to prevent elaborating on the previous stimulus and facilitate capturing the automatic rather than a reflective attention process [3]. The probe was two dots presented either vertically (“:”; in 50% of trials) or horizontally (“..”; 50% of trials) and participants were required to react as quickly and accurately as possible to the probe orientation by pressing the “Y” key for vertical or the ”M” key for horizontal orientation. The probe was followed by a blank screen presented for 1000 ms during which responses were recorded (within 1100ms after probe onset).

The task comprised four blocks in random order: two measuring AB for angry faces and two measuring AB for sad faces. Each block consisted of 20 congruent trials (i.e., emotional trials in which the probe appeared in the location of the emotional face), 20 incongruent trials (i.e., emotional trials in which the probe appeared in the location of the neutral face), and 10 neutral trials, summing up to a total of 40 congruent and 40 incongruent trials per emotion across the whole task. Within each block, trials were presented in random order with emotional faces as well as the probe presented equally often on each side. Before the first block, participants completed two practice blocks, each consisting of six trials in which they received feedback, in order to familiarize themselves with the task.

**Supplementary Figure 1.** Trial procedure of the modified Dot Probe Task (DPT; [1])

*
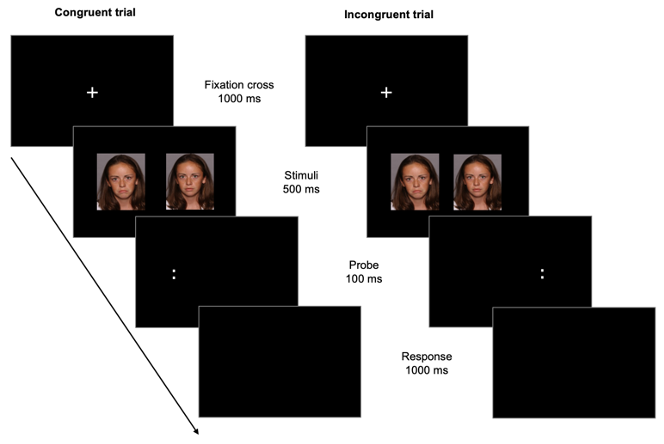
*

## Data Processing and outcome variables. In line with previous studies (e.g. [3]), trials with incorrect responses or reaction times (RT) shorter than 200 ms or longer than 2 SDs above each participant’s mean were excluded. Participants with less than 2 SD below the mean sum of correct trials of all participants were identified as outliers (in terms of response accuracy) and as such excluded from analysis (n = 2 MD children; n = 4 HR children, n = 1 LR child), resulting in a sample of 117 children for analysis of the DPT data. In the remaining sample, on average 164 correct and valid trials per participant were available for the children (SD = 17.5; 82% of 200 trials). This did not differ between groups (F < 1).

An AB score (AB_DPT_) was calculated by subtracting the mean RT in congruent trials from the mean RT in incongruent trials so that positive values indicate an AB towards negative information (see e.g. [3, 8]) while negative values indicate an AB away from negative information. AB_DPT_ scores for sad and angry faces were computed separately.

## Reliability. Split-half reliability of the task was assessed by correlating bias scores based on odd versus even trials (see e.g., [9]); it was poor for both emotions (angry: r = .01, p > .1; sad: r = .06, p > .1).

## Data analysis. One-way analyses of variance (ANOVAs) were conducted to assess group differences in AB_DPT_ scores. To assess relationships between psychopathology and AB, correlations were calculated between AB_DPT_ scores and depression as well as anxiety scores.

## Results. Bias scores were rather small for both angry and sad stimuli (see Supplementary table 3). The ANOVA revealed a significant effect of Group (F_2_ = 3.88; p = .023) in AB_DPT_ scores for angry faces, whereas no significant effect of Group (F_2_ = .53; p > .5) could be found regarding sad faces. For angry faces, there was a significant difference between HR and LR participants (t_82.8_ = 2.53; p = .014) with moderate effect size (d = 0.54), but not between HR and MD participants (ts ≤ 0.2; ps > .1) or LR and MD participants (ts ≤ 2.4; ps > .7). No significant correlations between AB_DPT_ scores and depression or anxiety scores were found (rs ≤ .13; ps > .1).

**Supplementary table 3.** AB_DPT_ scores for negative emotional faces (sad; angry) among all 3 groups.

|  | **MD** | **HR** | **LR** |
| --- | --- | --- | --- |
|  | *n* = 30 | *n* = 45 | *n* = 42 |
| AB_DPT_ for sad faces; *M (SD)* | 8.0 ms (26.1) | 1.4 ms (30.9) | 2.3 ms (27.1) |
| AB_DPT_ for angry faces; *M (SD)* | 0.6 ms (23.4) | 2.0 ms (30.4) | 12.8 ms (24.1) |

Note: AB_DPT_ = behavioural attention bias score from the Dot Probe Task; MD = major depression group; HR = high-risk group; LR = low-risk group; M = Mean; SD = Standard Deviation.

## Interpretation of results. The results indicate an AB towards angry faces in LR compared to HR youth but no group differences regarding AB for sad faces. However, due to the poor reliability of the outcome-parameters the interpretation is limited.

## References

1. Bradley, BP, Mogg, K, Falla, SJ, Hamilton, LR (1998). Attentional bias for threatening facial expressions in anxiety: Manipulation of stimulus duration. Cogn Emot 12(6):737-753.
2. MacLeod, C., Mathews, A., & Tata, P. (1986). Attentional bias in emotional disorders. J Abnorm Psychol 95(1):15.
3. Platt, B, Murphy, SE, Lau, JY (2015). The association between negative attention biases and symptoms of depression in a community sample of adolescents. PeerJ 3:e1372.
4. Schmukle, S. C. (2005). Unreliability of the dot probe task. Eur J Pers 19:595-605.
5. Waechter, S, Nelson, AL, Wright, C, Hyatt, A, Oakman, J (2014). Measuring attentional bias to threat: Reliability of dot probe and eye movement indices. Cognit Ther Res 38(3):313-333. <https://doi.org/10.1007/s10608-013-9588-2>
6. Platt, B, Sfärlea, A, Buhl, C, Loechner, J, Neumüller, J, Thomsen, LA et al. (2021). An Eye-Tracking Study of Attention Biases in Children at High Familial Risk for Depression and Their Parents with Depression. Child Psychiatry Hum Dev 1-20.
7. Egger, HL, Pine, DS, Nelson, E, Leibenluft, E, Ernst, M, Towbin, KE et al (2011). The NIMH Child Emotional Faces Picture Set (NIMH‐ChEFS): a new set of children's facial emotion stimuli. Int J Methods Psychiatr Res 20(3):145-156.
8. Klein, AM, de Voogd, L, Wiers, RW, Salemink, E (2018). Biases in attention and interpretation in adolescents with varying levels of anxiety and depression. Cogn Emot 32(7):1478-1486.
9. Van Bockstaele, B, Salemink, E, Bögels, SM, Wiers, RW (2017). Limited generalisation of changes in attentional bias following attentional bias modification with the visual probe task. Cogn Emot 31(2):369-376. <https://doi.org/10.1080/02699931.2015.1092418>

# Supplement 4: Scrambled Sentences Task

A computerized version of the Scrambled Sentences Task (SST; [1, 2]) was administered during eye-tracking (ET) in order to simultaneously assess attention biases (AB) to negative vs. positive words and interpretation biases, i.e. the tendency to form negative or positive statements out of ambiguous verbal information [1]. Interpretation bias results are reported elsewhere [3]. Due to the poor reliability we found in our previous study on AB in depressed parents and their offspring [4], we decided not to include this task in the manuscript.

## Stimuli. The stimuli consisted of 50 scrambled sentences, of which 30 were emotional sentences (e.g., “total I winner a loser am”) and 20 were neutral sentences (e.g., “like watching funny I exciting movies”). The emotional sentences were based on the original stimulus set developed by Wenzlaff and Bates [2] which was translated into German [5] adapted and extended. All sentences contained six words and had two possible solutions. In emotional trials, one solution was positive (e.g., “I am a total winner”) whereas the other was negative (e.g., “I am a total loser”). In neutral trials both solutions were emotionally neutral. Across the stimulus set, target words (the words in each sentence that accounted for the positive or negative solution) were matched for length and frequency in the German language.^[[1]](#footnote-1)^ In line with Everaert and colleagues [1], word position within each sentence was randomised, with target words not allowed next to each other or in the first or last position and counterbalanced whether the positive or negative target word was presented first.

## Task Procedure. Supplementary figure 2 depicts the trial procedure. Each trial began with a drift correction (small white circle on the left side of the screen) to direct the attention to that initial position. On fixation of the circle, the experimenter initiated the trial. A fixation cross followed for 500ms on the left side of the screen, preceding the stimulus display consisting of six words in a scrambled order presented at the centre of the screen on a single line. Participants were instructed to read the words and mentally form a grammatically correct five-word sentence as quickly as possible and to click on the mouse button as soon as they did so to continue to the response part of the trial. A scrambled sentence was presented for a maximum of 8000ms, if no mouse click occurred during that time, the response part was omitted and the next trial began. In the response part five boxes appeared below the scrambled sentence and participants were required to build the sentence they had mentally formed by clicking on the words in corresponding order. Time to allocate the words to boxes was not limited.

Trials were randomly divided into five blocks of ten (each containing six emotional and four neutral trials presented in random order). Before the first block participants completed five practice trials (five randomly selected neutral sentences) to familiarize themselves with the task.

Similar to earlier studies (e.g., [1, 6]) we added a cognitive load procedure to prevent deliberate report strategies. Before each block, a 6-digit number was presented to the parents and a 4-digit number was presented to the children for 5000ms. Participants were instructed to memorize the number in order to recall it at the end of the block.

**Supplementary figure 2**. Trial procedure of the Scrambled Sentences Task (SST; [1, 2])

*
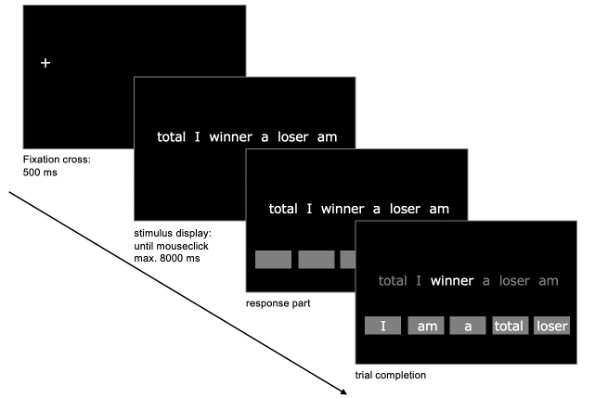
*

## Data Processing and outcome variables. Participants’ responses were rated as correct or incorrect. Trials in which no grammatically correct sentence was built (time-out or incorrect sentence) were excluded from the analysis. Participants with a correct sentence rate of two standard-deviations below the mean were identified as outliers in terms of accuracy and excluded (n=3 MD; n=8 HR; n=6 LR children). For the analyses of ET data we additionally excluded trials with poor data quality (see data processing of the Passive Viewing Task ET data in the manuscript for criteria) and participants with insufficient trials available due to data quality (MD: n = 23; HR: n = 37; LR: n = 34). In the remaining sample (94 children), on average 25.4 (SD = 2.68; 50.8% of 50 trials) trials per participant were available. This did not differ between groups (F_2,91_ = .29; p = .75; η^2^ = .01). To assess AB, we calculated an AB_SST_ score by dividing the percentage of dwell time on negative target words by the sum of percentages of dwell times on negative and positive target words, with higher values indicating a more negative AB [1].

## Reliability. Split-half reliability was assessed by correlating bias scores based on odd versus even trials (see e.g., [7]). Reliability was poor (r = -.17; p = .05).

## Data analysis. A one-way analysis of variance (ANOVAs) was conducted to assess group differences in AB_SST_ scores. To assess relationships between psychopathology and AB, correlations were calculated between AB_SST_ scores and depression as well as anxiety scores.

## Results. The one-way ANOVA revealed that AB_SST_ scores (MD: M (SD) = .51 (.04); HR: M (SD) = .49 (.04); LR: M (SD) = .50 (.06)) did not differ significantly between groups (F_2,91_ =1.42; p = .25; η^2^ = .03). No significant correlations emerged between AB_SST_ scores and depression or anxiety scores (rs < .1; ps > .35).

## Interpretation of results. The results show no evidence for group differences in AB between the groups. However, due to the poor reliability of the outcome-measure the interpretation is limited.

## References

1. Everaert, J, Duyck, W, Koster, EH (2014). Attention, interpretation, and memory biases in subclinical depression: A proof-of-principle test of the combined cognitive biases hypothesis. Emotion 14(2):331.
2. Wenzlaff, RM, Bates, DE (1998). Unmasking a cognitive vulnerability to depression: how lapses in mental control reveal depressive thinking. J Pers Soc Psychol, 75(6):1559.
3. Sfärlea, A, Buhl, C, Loechner, J, Neumüller, J, Thomsen, LA, Starman, K, et al. (2020). “I Am a Total… Loser”–The Role of Interpretation Biases in Youth Depression. J Abnorm Child Psychol 48(10):1337-1350.
4. Platt, B, Sfärlea, A, Buhl, C, Loechner, J, Neumüller, J, Thomsen, LA et al. (2021). An Eye-Tracking Study of Attention Biases in Children at High Familial Risk for Depression and Their Parents with Depression. Child Psychiatry Hum Dev 1-20.
5. Rohrbacher, H (2016). Interpretation bias in the context of depressed mood: Assessment strategies and the role of self-generation in cognitive bias modification.
6. Rude, SS, Valdez, C, Odom, S, Ebrahimi, A (2003). Negative cognitive biases predict subsequent depression. Cognit Ther Res 27(4):415-429.
7. Van Bockstaele, B, Salemink, E, Bögels, SM, Wiers, RW (2017). Limited generalisation of changes in attentional bias following attentional bias modification with the visual probe task. Cogn Emot 31(2):369-376.

# Supplement 5: Mood induction

Mood was assessed five times during the experimental session (see [1]) using the valence dimension of the 9-point Self-Assessment Mannequin scale [2] and analysed with a Time (5) × Group (3) ANOVA and subsequent t-tests.

## Results

The ANOVA yielded significant main effects of Time (F_2.1_ = 54.57; p < .001) and Group (F_2_ = 60.20; p < .001), resulting from the MD group reporting more negative mood than the HR (t_47.3_ = 8.3, p < .001) and LR groups (t_44.6_ = 8.5, p < .001), as well as a significant interaction of Time × Group (F_4.2_ = 8.89; p < .001). A post-hoc t-test comparing the baseline mood (mood before first mood induction: Time 1) with the other mood assessments revealed that participants rated their mood worse after watching the sad movie scene (Time 2 and Time 3; ts ≥ 7.82; ps < .001) and better after watching the pleasant movie scene (Time 5; t_122_ = 3.06; p = .003) compared to baseline. Since Time and Group interacted significantly, we conducted follow-up ANOVAs with the factor Time within all groups: in both healthy control groups, the effects of mood induction were significant (Fs < 43.22; p < .001), but not in the depressed group (F_2.4_ = 1.15; p = .33). This indicated that the mood induction only influenced the mood of the non-depressed participants but had no effect on the mood of participants with MD, since their mood was more negative beforehand and remained so throughout the experimental session (see Supplementary Figure 3).

We further conducted post-hoc t-tests comparing baseline mood (mood before first mood induction: Time 1) with the other mood assessments in the HR and LR groups. These analyses revealed that both HR as well as LR participants rated their mood as worse after watching the sad movie scene (Time 2 and Time 3; ts ≥ 5.3; ps < .001) and as better after watching the pleasant movie scene (Time 5; ts ≥ 2.1; ps ≤ .044) compared to baseline.

**Supplementary Figure 3.** Mean mood ratings of all children among groups in all 5 mood assessments.


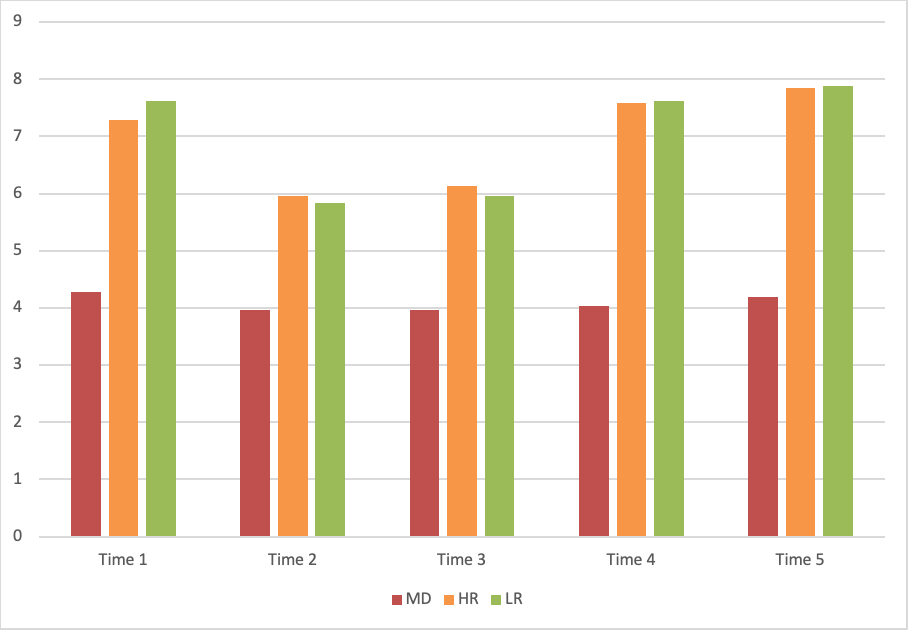


## References

1. Sfärlea, A, Löchner, J, Neumüller, J, Asperud Thomsen, L, Starman, K, Salemink, E, et al. (2019). Passing on the half-empty glass: A transgenerational study of interpretation biases in children at risk for depression and their parents with depression. J Abnorm Psychol 128(2):151-161. <https://doi.org/10.1037/abn0000401>
2. Lang, P. (1980). Behavioral treatment and bio-behavioral assessment: Computer applications. Technology in mental health care delivery systems 119-137, Ablex, Norwood, NJ, USA.

1. Positive target words: Word length *M* = 7.2 (*SD* = 2.5) characters, word frequency (category according to http://wortschatz.uni-leipzig.de/de) *M* = 9.8 (*SD* = 2.9); Negative target words: word length *M* = 7.2 (*SD* = 2.7) characters, word frequency *M* = 9.9 (*SD* = 4.1); *t*s <1 in paired t-tests. [↑](#footnote-ref-1)
